# The Association Between Neuroticism and Problematic Social Networking Sites Use: The Role of Fear of Missing out and Self-Control

**DOI:** 10.1177/00332941221142003

**Published:** 2022-11-25

**Authors:** Nino Gugushvili, Karin Täht, Eva Maria Schruff-Lim, Robert AC Ruiter, Philippe Verduyn

**Affiliations:** Department of Work and Social Psychology, 5211 Maastricht University, Maastricht, Netherlands; Department of Individual and Social Psychology, 37546 University of Tartu, Tartu, Estonia; Department of Individual and Social Psychology, 37546 University of Tartu, Tartu, Estonia; Institute of Mathematics and Statistics, 37546 University of Tartu, Estonia; Marketing and Consumer Behaviour Group, 4508 Wageningen University and Research, Netherlands; Department of Work and Social Psychology, 5211 Maastricht University, Maastricht, Netherlands

**Keywords:** problematic social networking sites use, neuroticism, fear of missing out, self-control, parallel mediation

## Abstract

Problematic use of social networking sites (SNS) has a negative impact on mental health. It has been found that people who score high on neuroticism are especially vulnerable towards engaging with SNS in a problematic way but it is not clear which psychological mechanisms explain this relationship. We addressed this issue by examining the mediating role of fear of missing out and self-control in the relationship between neuroticism and problematic SNS use. For this purpose, we conducted a cross-sectional study (*n* = 151, 69.5% female, M_age_ = 26.23, SD = 7.52) and tested for parallel mediation using structural equation modelling. Neuroticism was found to be predictive of increased levels of problematic SNS use. Moreover, neuroticism was associated with both increased levels of fear of missing out and decreased levels of self-control. However, only fear of missing out was found to robustly mediate the relationship between neuroticism and problematic use of SNS. These findings suggest that fear of missing out could be an intervention target to prevent people scoring high on neuroticism from engaging in problematic SNS use.

## Introduction

Social networking sites (SNS) have changed how people interact and play a major role in today’s society. During the past two decades, many SNS platforms have been developed, including Facebook, WeChat, TikTok, Twitter, and Instagram. SNS offer their users many benefits. For example, SNS allow people to present themselves (Hollenbaugh, 2021; Seidman, 2013) and communicate with others (Bayer et al., 2020). Moreover, SNS offer entertainment (Apaolaza et al., 2014), access to information (Asghar, 2015), and allow their users to build and feel part of a community (Blight et al., 2017; Ellison et al., 2007; Gao et al., 2017). People are aware of these benefits and SNS are therefore highly popular. Currently, more than half of the world’s population uses SNS and spends on average 2 hours and 25 minutes on these platforms each day (Chaffey, 2021).

Despite the many benefits SNS may offer, SNS also have the potential to negatively impact well-being (for meta-analytic evidence, see Ivie et al., 2020; Yoon et al., 2019). This is especially the case when SNS usage turns into a problematic or addiction-like behavior (Kuss & Griffiths, 2017). There is a general consensus that problematic SNS usage is a significant public health problem when it involves excessive SNS use that interferes with important life domains including work, studies, and leisure (Andreassen, 2015).

The Interaction of Person-Affect-Cognition-Execution model (I-PACE) (Brand et al., 2016, 2019) explains how problematic and addictive behaviors online are developed and maintained. According to this model, personality traits are among the most important predisposing factors which both directly and indirectly impact problematic usage of technologies. Wegmann and Brand (2019) further hypothesize that personality traits characterized by low social competence and social deficits are key drivers of excessive compensatory use of SNS. In this regard, neuroticism, characterized by frequent experiences of loneliness, feelings of personal inadequacy, inferiority, and elevated sensitivity to social threats (Denissen & Penke, 2008; McCrae & John, 1992; Watson et al., 1994), has been shown to be a key predictor of problematic SNS use (for meta-analytic evidence, see Marciano et al., 2020). Specifically, among the big five personality traits, neuroticism is the strongest predictor of problematic SNS use (for meta-analysis, see Huang, 2022). However, it is not fully clear which mechanisms account for this relationship.

The I-PACE model (Brand et al., 2016, 2019) further states that the effect of predisposing factors (e.g., neuroticism) on problematic usage of SNS can be mediated by multiple emotional and cognitive responses simultaneously. Fear of missing out (FoMO) which is defined as “a pervasive apprehension that others might be having rewarding experiences from which one is absent” (Przybylski et al., 2013, p. 1841) is thought to be a key negative reinforcing mechanism in this context (Wegmann & Brand, 2019). Specifically, according to the Fear Driven/Compensation Seeking Hypothesis (Wegmann & Brand, 2019), users with low social competence experience higher FoMO. Thus, they resort to excessive use of SNS in order to reduce FoMO and gratify social needs. In line with this reasoning, past studies show that FoMO mediates the relationship between psycho-social variables, including personality traits, and problematic usage of digital technologies (Oberst et al., 2017; Reer et al., 2019; Wegmann et al., 2017).

In addition to fear of missing out, other emotional and cognitive responses may mediate the relationship between neuroticism and problematic SNS use (Brand et al., 2019; Wegmann & Brand, 2019). As such, these reinforcing mechanisms may act as parallel but different routes when connecting personality traits with problematic usage patterns of digital technology. One such potential underlying mechanism pertains to self-control. Self-control is “the ability to override or change one’s inner response, as well as to interrupt undesired behavioral tendencies (such as impulses) and refrain from acting on them” (Tangney et al., 2004, p. 274).

Individuals with high neuroticism frequently experience negative mood, loneliness, and depression (Watson et al., 1994), and turn to SNS for mood regulation (Marino et al., 2016). However, according to the cognitive-behavioral model of generalized problematic internet use (Caplan, 2010), usage of internet applications for mood regulation causes failures in self-control. Furthermore, experiencing negative emotions also directly decreases self-control (Heatherton & Wagner, 2011; Schmeichel & Tang, 2015). In turn, decreased self-control (e.g., reckless behavior and acting without thinking) leads to problematic usage patterns of technology (Błachnio & Przepiorka, 2016; Cudo et al., 2020; Turel & Qahri-Saremi, 2016) because usage behavior is driven by immediate gratification (Slater, 2003).

This pattern of findings suggests that the predictive effect of neuroticism on problematic SNS use could be explained by high levels of FOMO and low levels of self-control. However, these two explanatory mechanisms of the relationship between neuroticism and problematic SNS use have never been tested in a single integrated empirical model.

The present study aims to simultaneously test the role of FOMO and self-control as mechanisms explaining the link between neuroticism and problematic SNS use. Given that problematic usage of SNS is fairly prevalent (Alzougool, 2018; Mamun & Griffiths, 2019; Turel et al., 2018), this will increase our understanding of the relationship between personality traits and problematic SNS use. Furthermore, this will also reveal potential mechanisms for interventions.

In the next sections, we first clarify the constructs problematic SNS use and neuroticism, and subsequently describe prior research on their interrelation. Next, we define the constructs FOMO and self-control, and summarize prior empirical research that suggests that these mechanisms may explain the relationship between neuroticism and problematic SNS use. Finally, we describe the theoretical frameworks underlying the present study and describe the hypotheses of the present study.

### Problematic SNS use

Problematic SNS use has been conceptualized within a behavioral addiction framework (Griffiths, 2005) and consists of six core components: (a) *salience* which implies that SNS usage becomes one’s central activity and constantly occupies one’s mind, (b) *mood modification* which implies that one uses SNS to alter negative emotional states, (c) *tolerance* which refers to the need to increase the amount of SNS usage to obtain former levels of pleasure derived from the same activity, (d) *withdrawal* which implies that reducing the amount of time spent on SNS leads to significant distress, (e) *relapse* which pertains to an inability to reduce SNS use, and (f) *conflict* which refers to interpersonal conflicts in the domains of work, studies, leisure or hobbies caused by excessive SNS use (Andreassen, 2015).

There is no consensus on which term is most optimally suited to cover excessive usage of SNS (Billieux et al., 2015a, 2015b). While some authors prefer the term “SNS addiction” (Abbasi, 2019; Andreassen, 2015; Blackwell et al., 2017), others avoid possible over-pathologization and prefer the term “problematic SNS use” (Boer et al., 2020; Huang, 2020; Hussain & Griffiths, 2018). In this paper, we will use the term problematic SNS after Panova and Carbonell (2018), who argue that problematic patterns of technology usage may represent a milder form of behavioral addictions. Moreover, the fact that SNS addiction has not (yet) been officially recognized as a psychiatric disorder is another reason to adopt the term problematic SNS use.

While there is some disagreement on the most optimal term to describe the phenomenon, there is a general consensus that problematic usage of SNS is a major pervasive problem. According to a recent meta-analysis, prevalence estimates of problematic SNS use range from 14% (individualistic nations) to 31% (collectivist nations) (Cheng et al., 2021). Moreover, problematic SNS use has been shown to be associated with many negative outcomes, including task distraction (Moqbel & Kock, 2018), impaired academic performance (Al-Menayes, 2015), romantic disengagement (Abbasi, 2018), impaired subjective well-being (for meta-analysis, see Huang, 2020) and psychiatric disorders (for a review, see Hussain & Griffiths, 2018). Consequently, there is a growing public concern about the impact of problematic SNS use on today’s society (Andersson, 2018).

### Neuroticism

Neuroticism is one of the central personality traits of the big-five taxonomy (McCrae & John, 1992) and refers to a lack of emotional stability and frequent experiences of negative emotions such as anger, frustration, worry, and anxiety. Neuroticism has been found to be predictive of a host of negative outcomes. Specifically, neuroticism is associated with impaired mental and physical health, lowered quality of life (Lahey, 2009), interaction anxiety (Newby et al., 2017), low social support (Lahey, 2009), and difficulties in relationships (McNulty, 2008). Furthermore, individuals with high levels of neuroticism frequently use maladaptive coping strategies including wishful thinking, withdrawal, denial, and substance use (Carver & Connor-Smith, 2010).

In the context of social media use, it has been shown that neurotic users gratify multiple social needs online. For instance, they use online settings for self-presentation (Seidman, 2013) and tend to express their true selves (Amichai-Hamburger et al., 2002; Tosun & Lajunen, 2010). Furthermore, users with high neuroticism prefer to engage in online communication as compared to face-to-face interactions (Abbasi, 2018) and compensate for feelings of belongingness (Seidman, 2013) and derive social support on SNS (Shen et al., 2015). However, compensation for these social needs through SNS increases the risk of SNS addiction (Marengo et al., 2020). As such, there is strong evidence for a positive relationship between neuroticism and problematic SNS use (Marciano et al., 2020). Nevertheless, there is a need to identify robust mediators of this relationship.

### Fear of Missing out Explaining the Relation Between Neuroticism and Problematic SNS use

When individuals experience FoMO, they want to stay up to date and continuously check what others are doing (Przybylski et al., 2013). FoMO is characterized by negative emotional experiences (affective component) and worry and rumination (cognitive component) (Elhai et al., 2021; Neumann, 2020; Przybylski et al., 2013; Wegmann et al., 2017). FoMO can occur in offline contexts as well but SNS provide an especially fertile ground for these experiences to take place by making social information easily accessible for users and offering an effortless way to stay constantly connected and keep tabs on what others are doing (Elhai et al., 2021).

Empirical evidence confirms that FoMO is positively associated with problematic SNS use and the magnitude of this relationship ranges from a medium (Fioravanti et al., 2021; Yali et al., 2021) to large effect size (Elhai et al., 2021). Furthermore, past research revealed that FoMO and neuroticism are separate (Rozgonjuk et al., 2021) but positively correlated constructs (Fioravanti et al., 2021).

Surprisingly, only one study directly examined the mediating role of FoMO in the relationship between neuroticism and problematic SNS use (Sindermann et al., 2021). Specifically, it has been demonstrated that FoMO mediates the association between neuroticism and problematic use of WeChat (Sindermann et al., 2021). However, it is unclear whether findings on problematic WeChat use hold for problematic SNS use in general. Moreover, FoMO was studied as a single mediating mechanism, ignoring the role of other possible key mechanisms such as self-control. This is troublesome as multiple mechanisms may be responsible for the association between neuroticism and problematic SNS use.

### Self-Control Explaining the Relation Between Neuroticism and Problematic SNS Use

Self-control is associated with a wide range of desirable outcomes, such as academic performance (Duckworth & Seligman, 2005), interpersonal success, low levels of psychopathology (Tangney et al., 2004), and increased well-being (de Ridder et al., 2012). Consistently, low levels of self-control have been found to be associated with undesirable outcomes, including impaired physical health (Miller et al., 2011), low income (Fergusson et al., 2013), and criminal and deviant behavior (Vazsonyi et al., 2017).

In the context of social media, self-control has been found to be related to problematic SNS use (for a review, see Zahrai et al., 2022). Specifically, high levels of self-control protect users from overusing social networking sites (Brevers & Turel, 2019), while deficiencies in self-control are related to problematic SNS use (Wu et al., 2015). In addition to impulsive behavior, which is aimed at immediate gratification (Duckworth & Steinberg, 2015), low self-control also implies having low self-discipline and having a hard time to break bad habits (Tangney et al., 2004), which may further contribute to maintaining problematic SNS usage patterns.

Given that users with high neuroticism have lower levels of self-control (Fetterman et al., 2010; Tangney et al., 2004), self-control might explain the relationship between neuroticism and problematic SNS use. Surprisingly, while it has been found that low levels of self-control mediate the relationship between loneliness and internet addiction (Özdemir et al., 2014), no study has examined the possible mediating role of self-control in the relationship between neuroticism and problematic SNS use. Furthermore, no study has examined whether FoMO and self-control parallelly mediate the relationship between neuroticism and problematic SNS use.

### Theoretical Frameworks on the Relationship Between Neuroticism, FoMO, Self-Control and Problematic SNS Use

We rely on the I-PACE model (Brand et al., 2019), the Fear Driven/Compensation Seeking Hypothesis (Wegmann & Brand, 2019), and the cognitive-behavioral model of generalized problematic internet use (Caplan, 2010) to explain the direct and indirect relationships between neuroticism and problematic SNS use. Specifically, according to the I-PACE model, the P component pertains to person-specific characteristics, such as temperamental features, genetics, and psychopathology (e.g., depression, social anxiety) which serve as vulnerability factors and precursors of different types of digital addictions. As such, they trigger specific affective (A component) and cognitive (C component) responses. While these responses and corresponding behaviors provide relief and gratification of needs (e.g., mood management, compensation of social deficiencies), they eventually lead to habitual and problematic usage patterns. Neuroticism can be assumed to be a core vulnerability factor in this context.

Moreover, fear of missing out may act as a mediating mechanism through which neuroticism is associated with problematic SNS use. This is in line with the Fear Driven/Compensation Seeking Hypothesis, which posits that users with low social competence and social deficiencies, as is the case for neurotic users, engage in SNS use to satisfy their social needs and deal with their feelings of FoMO. In turn, social gratification, and reduction of FoMO reinforces usage patterns and leads to problematic SNS use.

Besides fear of missing out, self-control may also mediate the relationship between neuroticism and problematic SNS use. In addition to social need satisfaction, mood regulation (e.g., reduction of negative affect) (Chen & Roberts, 2019) is another main motivation for neurotic users to engage with SNS. The cognitive-behavioral model of generalized problematic internet use (Caplan, 2010), however, maintains that usage of internet applications for mood management, as well as preference for online interaction leads to self-control failure, which in turn leads to problematic usage patterns. Based on this, (low) self-control may act as a parallel mechanism through which neuroticism is associated with problematic SNS use.

### The Present Study

The aim of this study is to shed light on the relationship between neuroticism and problematic SNS use by simultaneously testing the mediating role of FOMO and self-control. To our knowledge, no study has examined these mediating mechanisms together in a single model. To address this gap, we aim to account for the relationship between FOMO and self-control when examining their unique contribution in explaining the association between neuroticism and problematic SNS use. As problematic usage of SNS is quite prevalent, (Alzougool, 2018; Mamun & Griffiths, 2019; Turel et al., 2018), this will not only enhance our fundamental understanding of the relationship between personality traits and problematic SNS use but also pinpoint mechanisms that may be targeted by interventions to protect subpopulations of SNS users from engaging in problematic SNS use and associated declines in well-being. Moreover, the findings of this study are relevant for counsellors in order to evaluate and address excessive usage patterns of SNS among neurotic clients.

**Figure 1.**
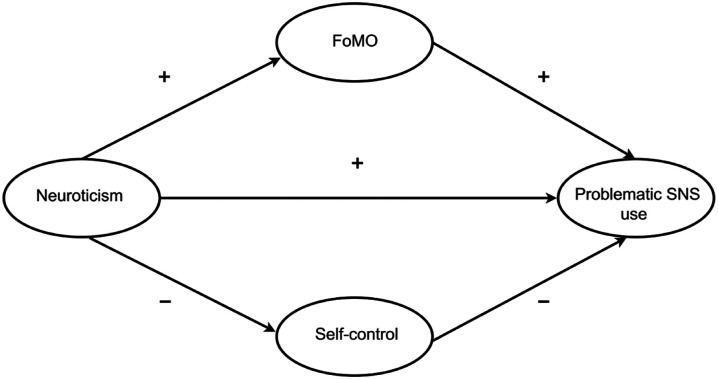
Conceptual Model of the Relation Between Neuroticism and Problematic SNS use Mediated by Fear of Missing Out and Self-control.

The conceptual model of the present study is displayed in Figure 1. We formulated the following hypotheses:

**H1**: *Fear of missing out mediates the relationship between Neuroticism and Problematic SNS use.* This mediation pathway has never been directly tested but it is consistent with prior research showing that FoMO mediates the positive relationship between neuroticism and problematic WeChat use (Sindermann et al. (2021).

**H2**: *Self-control mediates the relationship between Neuroticism and Problematic SNS use.* This relationship has never been tested. However, past studies reveal that neuroticism is negatively related to self-control (Mao et al., 2018) and self-control is negatively associated with problematic SNS use (Brevers & Turel, 2019).

**H3**: *Fear of missing out and self-control have an independent explanatory effect when examined as parallel mediators of the relationship between neuroticism and problematic SNS use.* FoMO and self-control are separate constructs that are differentially related to problematic SNS use. Therefore, we expect both construct to independently contribute to explaining the relationship between neuroticism and problematic SNS use.

## Method

### Participants

To recruit participants, we made use of a convenience sampling approach. Specifically, the study was advertised by posting a flyer of the study on several social media platforms (e.g., Facebook). Moreover, a research assistant contacted people in her social network asking them to participate in the study. The advantage of such a convenience sampling approach is that it allows to recruit participants in an efficient manner (Acharya et al., 2013). Participants were included if (a) they were 18 years or older and (b) used SNS. The latter was measured by asking participants to indicate: (1) whether they use SNS, but also (2) how much time they spent daily on SNS, and (3) which SNS apps they used most frequently. In total, 151 individuals (69.5% female) volunteered and participated in our study. Their age ranged from 18 to 64 (average age = 28.26, SD age = 7.52). All participants reported having a SNS account. Most participants were German (49%), followed by “Other” (41%), and Dutch (10%). Most participants used SNS for 1–3 hours per day (46%), while others mentioned using SNS each day for 30 minutes to 1 hour (30%), less than 30 minutes (11%) or 3–5 hours (10%). Moreover, most participants used Facebook (77%), followed by Instagram (68%), YouTube (66%), LinkedIn (42%), and Snapchat (33%). The study complied with research ethical guidelines and was approved by the Ethics Review Committee of Maastricht University, Ethics Review Committee Code: 161_03_02_2016.

Furthermore, we conducted a sensitivity analysis to detect the range of effect sizes (two-tailed correlation) that our study could detect reliably, given our sample size, α = .05, and 80% power. The results show that our study design is sufficiently powered to detect effect sizes of |p| = .23 and higher, which corresponds with prior research on the relationship between the constructs under consideration where typically correlations higher than .25 were reported (e.g., Balta et al., 2020; Błachnio & Przepiorka, 2016; Dempsey et al., 2019; Mao et al., 2018).

### Procedure and Materials

Upon providing informed consent, participants completed an online questionnaire. This questionnaire consisted of demographic questions followed by a set of measurement scales including neuroticism, FoMO, self-control, and problematic SNS use. All questionnaires were fully completed by all participants.

#### Neuroticism

We measured neuroticism by the Big Five Inventory (BFI) by John and Srivastava (1999). This scale consists of eight items and asks participants to rate to what degree specific characteristics apply to them. Example items are: “Can be tense”, “Can be moody”, and “Is relaxed, handles stress well”. All items were rated on a five-point Likert scale ranging from 1 (strongly disagree) to 5 (strongly agree). The average score of the scale was computed across the eight items for each participant. Reverse-scored items were recoded such that higher scores on this scale reflect higher levels of neuroticism. The Cronbach’s alpha of this scale in the original study was .84 (John & Srivastava, 1999) and in the present study is .83.

#### Fear of Missing Out

To measure Fear of Missing Out, we used the Fear of Missing Out Scale (FOMOS) developed by Przybylski and colleagues (2013). This scale consists of 10 items such as “I fear others have more rewarding experiences than me” and “I get anxious when I don't know what my friends are up to”. Participants were instructed to answer whether, in general, these items reflect their everyday experiences. All 10 items were rated on a five-point Likert scale ranging from 1 (not at all true of me) to 5 (extremely true of me), and higher scores indicate higher levels of FoMO. The mean score of the scale was calculated across the 10 items for each participant. The Cronbach’s alpha for this scale in the original study was .87 (Przybylski et al., 2013) and in the present study is .84.

#### Self-Control

Self-control was measured by the Brief Self-control Scale (BBSC) (Tangney et al., 2004) which contains 13 items, such as “I am good at resisting temptation” and “I do certain things that are bad for me, if they are fun.” Respondents were instructed to answer to what extent each of the statements reflects how they typically are. All items were rated on a five-point Likert scale, ranging from 1 (strongly disagree) to 5 (strongly agree). We recoded reverse-scored items such that higher scores on this scale reflect higher levels of self-control and computed the mean score of the scale across all items. The Cronbach’s alpha of this measure in the original study was .89 (Tangney et al., 2004) and in this study is .83.

#### Problematic SNS Use

Problematic SNS use was measured by the six-item Bergen Social Media Addiction Scale (BSMAS) (Andreassen et al., 2017), which evaluates the six core aspects of addiction: salience, mood modification, conflict, withdrawal, tolerance, and relapse. Participants were instructed to answer how often a series of statements applied to them during the last year: e.g., “Felt an urge to use social network sites more and more” and “Spent a lot of time thinking about social network sites or planned use of social network sites”. All items were rated on a five-point Likert scale, ranging from 1 (very rarely) to 5 (very often). Higher scores on this scale reflect higher levels of problematic SNS use. The mean score of the scale was calculated across six items for all participants. The Cronbach’s alpha for this questionnaire in the original study was .88 (Andreassen et al., 2017), and in this study is.71.

### Statistical Approach

We used R (version 4.0.3) to analyze the data. We first computed descriptive statistics and bivariate correlations among the main variables. Next, we examined the factorial structure of our main variables using the R package Lavaan v.0.6–7 (Rosseel, 2012) which allows to conduct confirmatory factor analysis. We used standard parameters for judging the goodness of fit, including the Chi Square test, Comparative Fit Index (CFI), Tucker-Lewis Index (TLI), Root Mean Square Error of Approximation (RMSEA), and Standardized Root Mean Square Residual (SRMR) (Hu & Bentler, 1999; Kline, 2012).

Finally, to test our hypotheses, we constructed structural equation models in Lavaan. Specifically, we first built a single mediation model to test whether FoMO mediates the relationship between neuroticism and problematic SNS use when self-control is not accounted for. Second, we examined a single mediation model to test whether self-control mediates this relationship when FoMO is not accounted for. Third, we built a parallel mediation model to capture the effects of multiple mediators in a single integrated model. Compared to single mediation models, parallel mediation models “allow a variable’s effect to be transmitted to another through multiple mechanisms simultaneously” (Hayes, 2017, p. 147). Furthermore, in parallel mediation models, a specific indirect effect (e.g., the effect through FoMO) is estimated controlling for the other parallel mediators specified in the model. As such, by fitting a parallel mediation model we were able to take into account the correlation between FoMO and self-control (Hayes, 2017).

For the confirmatory factor analysis and mediation models, the bootstrapping technique (Preacher et al., 2007) was used across 1000 samples. Please note that both for confirmatory factor analysis and mediation models, we used Diagonally Weighted Least Squares estimation (DWLS) because this method is less biased for ordinal data (Mîndrilă, 2010). Moreover, when examining the relationship between our key constructs, we always controlled for the effect of gender and age on problematic SNS use.

## Results

### Descriptive Statistics and Correlations

Descriptive statistics for the main study variables, including the means, standard deviations, and correlations among them are displayed in the table 1. As expected, the relationship between neuroticism and problematic use of SNS was positive and significant.

**Table 1.** Descriptive Statistics and Correlations Between Age, Gender, SNS use frequency, Neuroticism, FoMO, Self-Control, and Problematic SNS Use.

Variable	M	SD	2	3	4	5	6	7
1. Age	28.26	7.52	−0.08	−0.17*	−0.19*	−0.37**	.29**	−0.32**
2. Gender^a^	1.71	.47		.05	.27**	.08	.02	.18*
3. SNS use frequency	2.67	.94			.21**	.32**	−0.15	.42**
4. Neuroticism	2.86	.77				.33**	−0.43**	.33**
5. FoMO	2.25	.70					−0.38**	.46**
6. Self-control	3.07	.68						−0.32**
7. Problematic SNS use	2.13	.66						

*Note*. *p < .05. **p < .01.

a 0 = male, 1 = female.

### The Structure of the Key Variables

Prior to testing our hypotheses, we examined the structure of our key variables (table 2). Specifically, we built four separate measurement models for neuroticism, FoMO, self-control, and problematic SNS use and utilized confirmatory factor analysis to check whether the hypothesized unidimensional models fitted our observed data well. All models except for self-control fitted data well. Specifically, the fourth item of the self-control scale, “I say inappropriate things,” had a suboptimal loading (< .3). Therefore, we reran CFA without this item. After implementing this change, all items loaded well on one latent variable (self-control). As can be seen in the table 2, all measurement models fitted the data well.

**Table 2.** Goodness-of-Fit Indicators of Models for Neuroticism, FoMO, Self-Control, and Problematic SNS Use.

Model	χ^2^ (df)	*p*	SRMR	TLI	CFI	RMSEA	90% CI	
							LL	UL
Neuroticism	13.460 (20)	.857	.054	1	1	.000	.000	.039
FoMO	43.712 (35)	.148	.077	.98	.98	.041	.000	.075
Self-control	53.352 (54)	.500	.070	1	1	.000	.000	.050
Problematic SNS use	9.611 (9)	.383	.057	.99	.99	.021	.000	.096
Mediation model: FMO	410.164 (295)	.000	.090	.95	.95	.051	.039	.063
Mediation model: Self-control	393.161(346)	.041	.085	.98	.98	.030	.007	.044
Mediation model: FoMO and self-control	784.843 (658)	.000	.087	.97	.97	.036	.025	.045

*Note*. CI = Confidence interval; LL = Lower limit; UL = Upper limit.

### Does FoMO Mediate the Relationship Between Neuroticism and Problematic SNS Use?

Next, we built a simple mediation model with structural equation modelling to test whether FoMO mediates the relationship between neuroticism and problematic SNS use. Neuroticism was found to positively predict FoMO (*B* = .625, β = .420, *SE* = .185, *p =* .001)*.* Moreover, FoMO positively predicted problematic SNS use (*B* = .360, β = .489, *SE* = .122, *p* = .003). Importantly, the indirect relationship between neuroticism and problematic SNS use through FoMO was significant (*B* = .225, β = .205, *SE* = .103, *p =* .029*,* 95% CI [.086, .481]). Moreover, the direct relationship between neuroticism and problematic SNS use was not significant (*B* = .255, β = .232, *SE* = .146, *p* = .080), further confirming the mediating role of FoMO. Finally, it is notable that the total relationship between neuroticism and problematic SNS use was significant (*B* = .480, β = .437, SE = .181, *p* = .008).

### Does Self-Control Mediate the Relationship Between Neuroticism and Problematic SNS Use?

We built a second simple mediation model with structural equation modelling to test whether self-control mediates the relationship between neuroticism and problematic SNS use. Neuroticism was found to be negatively related to self-control (*B* = -.272, β = -.520, *SE* = .097, *p = .*005*).* However, self-control did not significantly predict problematic SNS use (*B* = -.428, β = -.319, *SE* = .320, *p* = .181). Consequently, the indirect relationship between neuroticism and problematic SNS use through self-control was only marginally significant (*B* = .116, β = .166, *SE* = .067, *p = .081,* 95% CI [.022, .283])*.* The direct relationship between neuroticism and problematic SNS use was also still marginally significant (*B* = .201, β = .287, *SE* = .116, *p* = .082)*.* Finally, it is notable that the total relationship between neuroticism and problematic SNS use was statistically significant (*B* = .317, β = .453, *SE* = .124, *p* = .011)*.*

Are FoMO and self-control parallel mediators of the relationship between neuroticism and problematic SNS use?

**Figure 2.**
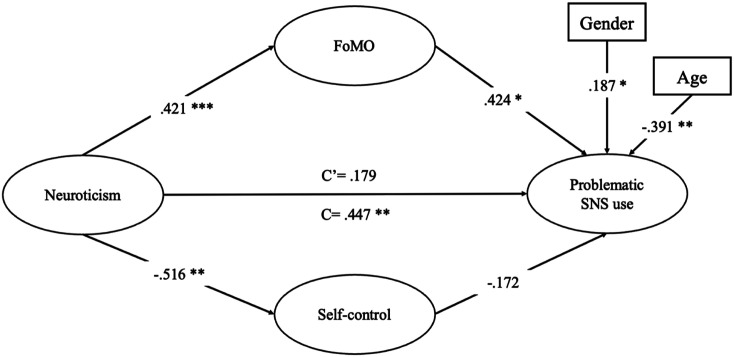
FoMO and Self-control as Parallel Mediators of the Relationship Between Neuroticism and problematic SNS use. *Note.* Regression weights are standardized. C’ is the direct effect of neuroticism on problematic SNS use. C is the total effect of neuroticism on problematic SNS use. Gender was coded as 0 = males, 1 = females. *p < .05; **p < .01; ***p < .001.

Finally, we built a parallel mediation model in Lavaan and examined whether the relationship between neuroticism and problematic SNS use was parallelly mediated via FoMO and self-control (table 3, Figure 2).

**Table 3.** Covariances, Direct, Indirect, and Total Effects of Neuroticism on Problematic SNS use.

Direct effects	B	β	SE	p
Gender → problematic SNS use	.243	.187	.123	.049
Age → problematic SNS use	−.031	−.391	.010	.001
Neuroticism → problematic SNS use	.162	.179	.126	.199
Neuroticism → FoMO	.567	.421	.151	<.001
Neuroticism → self-control	−.303	−.516	.097	.002
FoMO → problematic SNS use	.285	.424	.124	.021
Self-control → problematic SNS use	−.264	−.172	.299	.376
**Indirect effects (parallel mediation)**				
FoMO	.162	.179	.081	.045
Self-control	.080	.089	.072	.267
**Total effect**				
Neuroticism → problematic SNS use	.404	.447	.148	.006
**Covariances**				
FoMO - self-control	−.086	−.321	.036	.017
Gender - age	−.234	−.068	.248	.343

Neuroticism was found to be positively associated with FoMO (*B* = .567, β = .421, *SE* = .151, *p <* .001)*,* and negatively associated with self-control (*B* = -.303, β = -.516, *SE* = .097, *p = .*002). In turn, FoMO was positively associated with problematic SNS use (*B* = .285, β = .424, *SE* = .124, *p =* .021) but the relationship between self-control and problematic SNS use was not significant (*B* = -.264, β = -.172, *SE* = .299, *p = .*376). With regard to indirect effects, FoMO was found to be a significant mediator of the relationship between neuroticism and problematic SNS use (*B* = .162, β = .178, *SE* = .081, *p = .045,* 95% CI [.053, .368])*.* However, self-control did not mediate the relationship between neuroticism and problematic SNS use (*B* = .080, β = .089, *SE* = .072, *p = .267,* 95% CI [-.062, .226])*.* The direct relationship between neuroticism and problematic SNS use was not significant (*B* = .162, β = .179, *SE* = .126, *p =* .199), suggesting full mediation*.* Finally, it is notable that the total effect of neuroticism on problematic SNS use was significant (*B* = .404, β = .447, *SE* = .148, *p =* .006).

## Discussion

The aim of the present study was to examine the relationship between neuroticism and problematic SNS use by focusing on two underlying mechanisms that may explain this relationship: FoMO and self-control. First, we examined whether neuroticism predicts problematic SNS use and found this to be the case. This finding is consistent with the I-PACE model (Brand et al., 2019) which argues that maladaptive personality traits contribute to problematic usage patterns. Furthermore, this finding is also consistent with meta-analytic evidence revealing a consistent link between neuroticism and different types of problematic technology usage, including internet addiction (Kayiş et al., 2016), excessive use of smartphones, online gaming, and SNS (Marciano et al., 2020). More generally, our results are also consistent with prior research showing that people high in neuroticism engage in maladaptive coping strategies (Carver & Connor-Smith, 2010). However, we did not only describe the relationship between neuroticism and problematic SNS use but also attempted to explain it by examining the mediating role of FoMO and self-control.

Specifically, based on the I-PACE model (Brand et al., 2019) and the Fear Driven/Compensation Seeking Hypothesis (Wegmann & Brand, 2019), we expected that FoMO, which has both cognitive and affective components (Elhai et al., 2021; Przybylski et al., 2013; Wegmann et al., 2017) would act as a mediating mechanism in the relationship between neuroticism and problematic SNS use. In response to perceived social deficiencies, neurotic users would experience higher levels of FoMO (e.g., rumination, fear of exclusion) and turn to excessive usage of SNS to compensate for lack of social relationships and relieve FoMO. In line with this theoretical reasoning, we found that FoMO explains (mediates) the relationship between neuroticism and problematic SNS use, regardless of whether the effect of self-control is controlled for. This finding is consistent with the results of prior studies demonstrating a mediating role of FoMO in the relationship between maladaptive predisposing variables and excessive use of technologies (e.g., Dempsey et al., 2019; Wegmann et al., 2017). Moreover, while Sindermann and colleagues (2021) demonstrated that FoMO mediates the relationship between neuroticism and problematic WeChat use, we extended these findings by demonstrating that the explanatory role of FoMO is not restricted to WeChat but holds for SNS more generally.

Next, based on the cognitive-behavioural model of generalized problematic internet use (Caplan, 2010), which argues that usage of SNS for mood regulation and preference for online interactions leads to failures in self-control, we expected that neurotic users would attempt to regulate negative moods and experience self-control failures. In turn, decreased self-control would contribute to excessive usage of SNS. However, self-control was not found to be a robust mediator of the relationship between neuroticism and problematic SNS use. The indirect relationship between neuroticism and problematic SNS use through self-control was only marginally significant and turned non-significant when controlling for the mediation effect of FoMO. As such, FoMO rather than self-control seems to be the key mechanism explaining the relationship between neuroticism and problematic SNS use.

### Theoretical and Practical Implications

There is a growing public and scholarly concern that problematic SNS use has a negative impact on people’s well-being in today’s society. It is therefore of key importance to identify which populations are especially vulnerable to developing problematic SNS use. The present findings suggest that people who score high on neuroticism are at increased risk of developing problematic SNS use. This does not only enhance our fundamental understanding of user characteristics predicting problematic SNS use but also informs public health policy makers and counselors on which people are especially vulnerable towards developing problematic SNS use.

We also demonstrated that FoMO is a key mechanism connecting neuroticism to problematic SNS use. This finding increases our theoretical understanding of the mechanisms through which vulnerable populations may eventually develop problematic SNS use. Moreover, this finding may inform counselors how to help people high in neuroticism to engage with SNS in a healthy manner. Specifically, whereas interventions may not allow to fundamentally change someone’s personality, public health policy makers and counselors could focus their efforts on reducing FoMO to protect people from problematic SNS use. In this regard, the FoMO Reduction (FoMO-R) approach (Alutaybi et al., 2020) holds promising potential by offering specific strategies, such as using checklists and self-talk to reduce FoMO on SNS. Another promising avenue with regards to FoMO management pertains to mindfulness-based interventions (Weaver & Swank, 2021).

### Limitations and Future Research

The present study extended our understanding of the relationship between neuroticism and problematic SNS but a number of limitations should be noted. First, due to the cross-sectional design of the study, it is not possible to make conclusions on causal effects. Second, we measured problematic SNS use via self-report measures. Future research should consider using objective measures as more ecologically valid alternatives for measuring problematic usage of SNS (Ryding & Kuss, 2020). Third, the participant sample consisted mainly of young female SNS users. Given that female users are more inclined to engage in problematic SNS use (Su et al., 2020), research on problematic SNS use in women is highly important. However, future studies having a good gender balance are necessary, especially as males are typically underrepresented in social media research (Cheng et al., 2021). Moreover, future studies are needed to identify additional mechanisms which play a mediating role between neuroticism and problematic use of SNS. Lastly, our study focused on neuroticism which is associated with low social competence and negative emotions. However, Wegmann and colleagues (2019) suggest that socially integrated users are also at risk of developing problematic SNS usage patterns. These users can be reward-driven, and mechanisms such as positive feedback on SNS (e.g., likes) can positively reinforce excessive usage patterns. In this context, it would be relevant to investigate the predisposing role of extraversion because extraverted users are highly sociable and reward-seeking (Costa & McCrae, 1992), and often use SNS for pleasurable experiences (Chen & Roberts, 2019).

## Conclusion

In the present study, we found that people high in neuroticism more often suffer from problematic SNS use. Moreover, neuroticism was found to be related to both high levels of FoMO and low levels of self-control but only FoMO was found to be a robust mediator of the relationship between neuroticism and problematic SNS use. This suggest that FoMO might be a good intervention target to protect people from engaging in problematic SNS use and associated negative consequences.
